# Preoperative vascular heterogeneity based on dynamic susceptibility contrast MRI in predicting spatial pattern of locally recurrent high-grade gliomas

**DOI:** 10.1007/s00330-023-10149-6

**Published:** 2023-09-02

**Authors:** Hanwei Wang, Linlan Zeng, Hao Wu, Jing Tian, Huan Xie, Letian Zhang, Qisheng Ran, Peng Zhong, Lizhao Chen, Liang Yi, Shunan Wang

**Affiliations:** 1 Department of Radiology, Daping Hospital, Army Medical University, Chongqing, China; 2 Chongqing Clinical Research Center of Imaging and Nuclear Medicine, Chongqing, China; 3 Department of Pathology, Daping Hospital, Army Medical University, Chongqing, China; 4 Department of Neurosurgery, Daping Hospital, Army Medical University, Chongqing, China

**Keywords:** Glioma, Recurrence, Magnetic resonance imaging, Prognosis, Cerebral blood volume

## Abstract

**Objectives:**

To investigate if spatial recurrence pattern is associated with patient prognosis, and whether MRI vascular habitats can predict spatial pattern.

**Methods:**

In this retrospective study, 69 patients with locally recurrent high-grade gliomas (HGGs) were included. The cohort was divided into intra-resection cavity recurrence (ICR) and extra-resection cavity recurrence (ECR) patterns, according to the distance between the location of the recurrent tumor and the resection cavity or surgical region. Four vascular habitats, high angiogenic tumor, low angiogenic tumor, infiltrated peripheral edema, and vasogenic peripheral edema, were segmented and vascular heterogeneity parameters were analyzed. The survival and diagnostic performance under different spatial recurrence patterns were analyzed by Kaplan–Meier and ROC. A nomogram model was constructed by regression analysis and validated by bootstrapping technique.

**Results:**

Progression-free survival (PFS) and overall survival (OS) were longer for ICR (*n* = 32) than those for ECR (*n* = 37) (median PFS: 8 vs. 5 months, median OS: 17 vs. 13 months, *p* < 0.05). MRI vascular habitat analyses showed ECR had higher median relative cerebral blood volume (rCBV_median_) at each habitat than ICR (all *p* < 0.01). The rCBV_median_ at IPE had good diagnostic performance (AUC: 0.727, 95%CI: 0.607, 0.828). The AUC of the nomogram based on MRI vascular habitats and clinical factors was 0.834 (95%CI: 0.726, 0.913) and was confirmed as 0.833 (95%CI: 0.830, 0.836) by bootstrapping validation.

**Conclusions:**

The spatial pattern of locally recurrent HGGs is associated with prognosis. MRI vascular heterogeneity parameter could be used as a non-invasive imaging marker to predict spatial recurrence pattern.

**Clinical relevance statement:**

Vascular heterogeneity parameters based on MRI vascular habitat analyses can non-invasively predict the spatial patterns of locally recurrent high-grade gliomas, providing a new diagnostic basis for clinicians to develop the extent of surgical resection and postoperative radiotherapy planning.

**Key Points:**

*• Intra-resection cavity pattern was associated with longer progression-free survival and overall survival in locally recurrent high-grade gliomas.*

*• Higher vascular heterogeneities in extra-resection cavity recurrence than in intra-resection cavity recurrence and the vascular heterogeneity parameters had good diagnostic performance in discriminating spatial recurrence pattern.*

*• A nomogram model based on MRI vascular habitats and clinical factors had good performance in predicting spatial recurrence pattern.*

**Supplementary information:**

The online version contains supplementary material available at 10.1007/s00330-023-10149-6.

## Introduction

High-grade gliomas (HGGs, WHO grades III–IV) are common primary malignant tumors of the central nervous system in adults [1]. HGGs recur after 6 to 8 months in most patients despite standard therapy [2, 3].

**Fig. 1:**
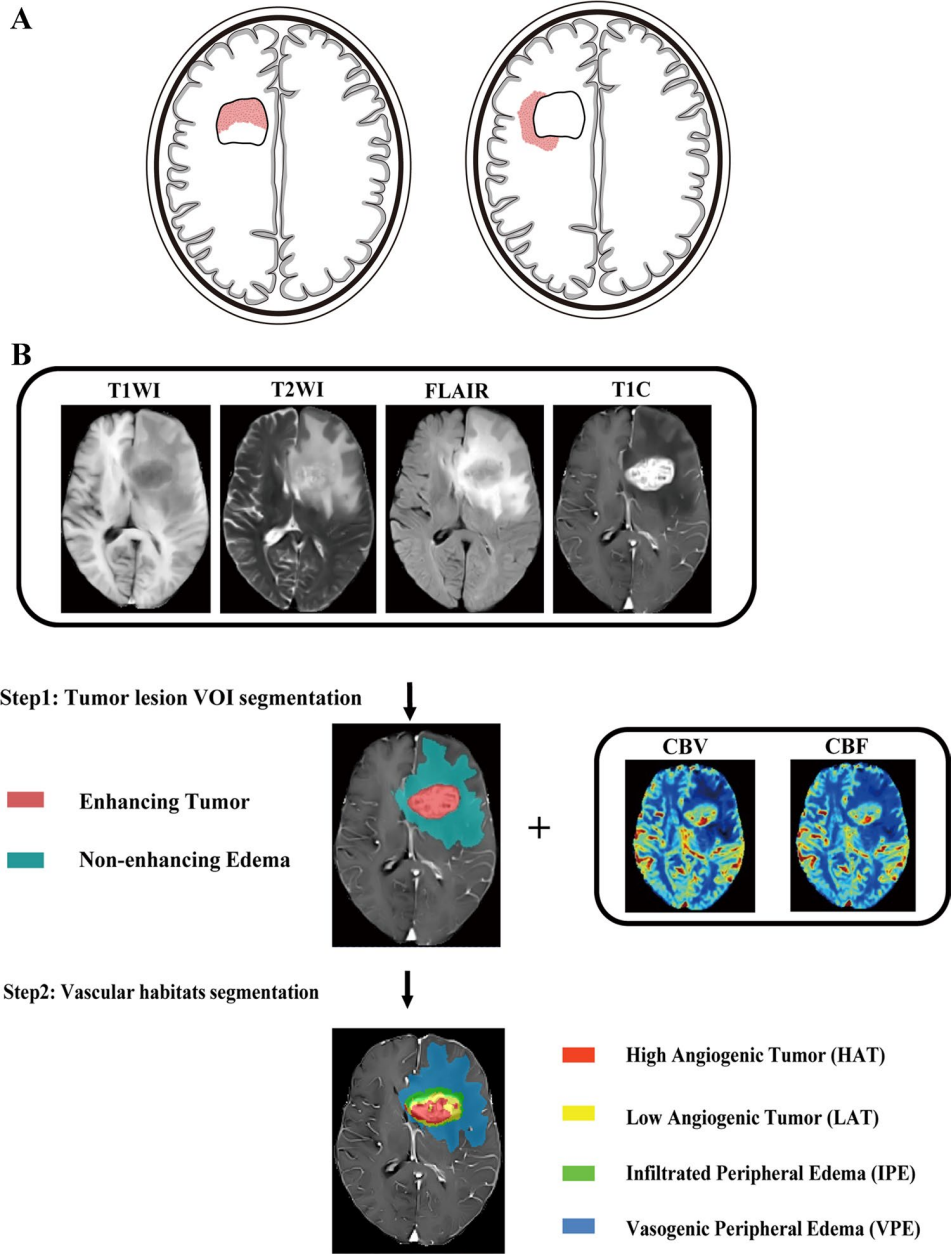
**A** Illustration of the spatial pattern of local recurrence, including intra-resection cavity recurrence versus extra-resection cavity recurrence (left to right). **B** The flowchart shows the image processing on vascular habitats. T1WI T1-weighted image, T2WI T2-weighted image, FLAIR fluid attenuation inversion recovery, T1C T1-weighted contrast-enhanced, VOI volume of interest, CBV cerebral blood volume, CBF cerebral blood flow

Local recurrence refers to recurrence within 2 cm of the tumor resection region, which is the most common glioma recurrence pattern, accounting for approximately 80% of all recurrent HGGs [4–6]. The treatment strategies differ for different recurrence patterns. For patients with local recurrence, individualized repeat surgical resection is generally recommended. In addition, the recurrence pattern of HGGs is associated with prognosis, and patients with local recurrence have better prognosis than those with non-local recurrence [7, 8]. Previous studies and clinical practice have suggested that local recurrence of HGGs has different spatial patterns, which can be divided into intra-resection cavity recurrence (ICR) and extra-resection cavity recurrence (ECR) based on the relationship between the resection cavity or surgical region and the recurrence tumor (Fig. 1A) [9]. However, it is still unclear whether the spatial pattern of locally recurrent HGGs is associated with prognosis. Meanwhile, there is a lack of studies on whether imaging markers can predict the spatial pattern of local recurrence.

Vascular habitats are an emerging MRI processing technique based on structural and dynamic susceptibility contrast (DSC) MRI to segment tumor subregions and non-invasively assess tumor vascular heterogeneity [10]. The vascular habitats utilize the hemodynamic tissue signature (HTS) method to segment tumors into four vascular habitats, namely high angiogenic tumor (HAT), low angiogenic tumor (LAT), infiltrated peripheral edema (IPE), and vasogenic peripheral edema (VPE) [10, 11], and calculate vascular heterogeneity parameters based on DSC. The vascular habitats can evaluate the prognosis and molecular subtyping of HGGs [12–16]. The findings of vascular habitats in predicting spatial recurrence pattern have not been reported in the literature as of yet.

Therefore, we aimed to investigate the prognosis of different spatial patterns of locally recurrent HGGs, and to determine whether MRI vascular habitats can predict the spatial pattern of locally recurrent HGGs.

## Materials and methods

### Study patients

This retrospective study was approved by the institutional review board of the Army Medical Center of the PLA, and written informed consent was waived. We analyzed the clinical, pathological, imaging, and survival data of patients with local recurrence of HGGs after maximum safe resection and chemoradiotherapy between January 2012 and December 2020.

The inclusion criteria were as follows: (1) histopathologic confirmation of newly diagnosed HGGs according to the 2016 WHO classification criteria, (2) no history of chemotherapy or radiotherapy treatment before surgery, (3) preoperative MRI, including T1-weighted imaging (T1WI), T2-weighted imaging (T2WI), fluid-attenuated inversion recovery (FLAIR) imaging, and contrast-enhanced (CE) T1-weighted imaging, and (4) age > 18 years. The exclusion criteria were as follows: (1) infratentorial HGGs, (2) low-quality MR images, including noise and artifacts, (3) patients with subarachnoid dissemination, (4) patient underwent subtotal resection, (5) patient not undergoing chemoradiotherapy after surgery, (6) patient lost to follow-up, (7) no recurrence during follow-up, (8) non-local recurrent HGGs, (9) patients did not undergo preoperative DSC sequences, and (10) errors when processing with HTS data processing pipeline.

### Image acquisition

MRI was performed with a 3.0-T MR scanner (Verio, Siemens Healthcare) with an 8-channel head coil. The parameters for the structural MRI sequences included (1) T1WI: repetition time (TR)/time of echo (TE), 250/2.67 ms; matrix, 320 × 256; slice thickness, 5 mm; and field of view (FOV), 230 mm × 230 mm; (2) T2WI: TR/TE, 4900/100 ms; matrix, 320 × 320; slice thickness, 5 mm; FOV, 230 mm × 230 mm; (3) FLAIR: TR/TE, 8000/94 ms; matrix, 256 × 256; slice thickness, 5 mm; FOV, 230 mm × 230 mm; inversion time (TI), 2370 ms; and (4) the parameters of CE-T1WI were consistent with those of T1WI. We performed CE-T1WI after DSC.

DSC was performed with a gradient-echo T2-weighted echo-planar imaging sequence during the injection of gadolinium contrast agent (gadopentetate glucosamine, Gd-DTPA; Beilu Pharmaceutical Co., Ltd.). The parameters for DSC perfusion imaging included TR/TE, 1500/30 ms; matrix, 128 × 128; slice thickness, 5 mm; FOV, 230 × 230; and flip angle, 90°. A bolus injection of 0.2 mL/kg of Gd-DTPA was administered at 4 mL/s by using a power injector. Before the eighth phase scan of the DSC, Gd-DTPA was injected, and followed by 15–20 mL saline. A total of 20 sections, 90 phases, and 1800 images were obtained.

### Image processing on vascular habitats

Preoperative MRI data were processed using the HTS service on the ONCOhabitats platform (https://www.oncohabitats.upv.es) [10, 17]. The MRI data processing flow was as follows: (1) preprocessing: correction of magnetic bias field inhomogeneities, noise, or spike artifacts, automated registration, brain extraction, and intensity normalization were performed to generate consistent multiparametric high-quality MRI of the brain; (2) segmentation: the tumor tissues were obtained using the art 3D convolutional neural network classifier based on a U-Net architecture; (3) DSC quantification: contrast material leakage correction was performed by means of gamma-variate curve fitting. Relative cerebral blood volume (rCBV) was performed by numerical integration of the area under the gamma-variate curve and relative cerebral blood flow (rCBF) was calculated based on the block-circulant singular value decomposition deconvolution scheme; (4) vascular habitats: the HTS method provided an automated unsupervised method to characterize tumor vascular heterogeneity. Tumors were segmented into four vascular habitats: HAT, LAT, IPE, and VPE. The flowchart of image processing on vascular habitats is shown in Fig. 1B. The median/mean/max rCBV (rCBV_median_/rCBV_mean_/rCBV_max_) and the median/mean/max rCBF (rCBF_median_/rCBF_mean_/rCBF_max_) were calculated for HAT, LAT, IPE, and VPE, respectively.

### Follow-up

All patients underwent maximum safe resection (all enhancing tumor removed or fluorescence-guided surgery with 5-aminolevulinic acid for resection enhancing tumor) and followed by concomitant radiotherapy combination with temozolomide and then adjuvant temozolomide. All patients underwent CE-MRI (and/or CT) within 24–72 h postoperatively, underwent CE-MRI 2–6 weeks after radiotherapy, and were then followed up with CE-MRI every 3–6 months. All patients were followed up until death or the study cut-off time (August 2022). Patients underwent reoperation, multi-disciplinary treatment, or continuous CE-MRI according to the Response Assessment in Neuro-Oncology (RANO) criteria to confirm recurrence, or multimodal MRI to rule out radiation necrosis or pseudoprogression. Progression-free survival (PFS) was calculated from the date of surgery to the first tumor recurrence on MRI. Overall survival (OS) was calculated from the date of surgery to death. Patients who survived until the last follow-up or cut-off time were considered censored.

### Evaluation of spatial recurrence pattern and radiological features

A radiologist and neurosurgeon (S.N.W. and L.Y., with 13 and 15 years of experience in brain imaging, respectively) evaluated the spatial recurrence pattern. The spatial recurrence pattern assessment process was as follows: (1) The surgical region was evaluated by CE-MRI and/or CT at 24–72 h postoperatively. The changes of the surgical region and the formation of resection cavity were observed during the follow-up CE-MRI. (2) The spatial pattern of locally recurrent HGGs was assessed based on the distance between the location of the recurrent tumor and the resection cavity or surgical region [9, 18]. The spatial pattern of local recurrence was divided into ICR and ECR. ICR was defined as recurrence located in the resection cavity or surgical region. ECR was defined as recurrence at the edge of or within 2 cm of the resection cavity or surgical region. (3) If the recurrence lesions were too extensive, the spatial recurrence pattern was determined based on the majority of the lesions located inside or outside the resection cavity or the surgical region and the growth trend of the recurrent lesions showed by continuous CE-MRI.

Cortex infiltration was defined as enhanced tumor involvement in the cerebral cortex. Subventricular zone (SVZ) involvement was defined as enhanced tumor contact with the lateral ventricular edge. Ventricular entry was defined as intraoperative access to the ventricle and confirmed by postoperative MRI that the ventricle was connected to the resection cavity. The above radiological features were assessed by a neurosurgeon and neuroradiologist (S.N.W. and L.Y.).

### Statistical analysis

Statistical analyses were performed using the Statistical Package for the Social Sciences for Windows (IBM SPSS Statistics, version 26.0, IBM Corp), MedCalc software (version 20.116, MedCalc Software Ltd.), GraphPad PRISM (version 9.0.0, GraphPad Software), and R software (version 4.2.1, R Foundation for Statistical Computing). A two-tailed significance level of *α* = 0.05.

Quantitative variables were presented as mean ± standard deviation. Categorical variables were expressed as percentages. Inter-rater reliability analysis of image assessment was evaluated by Cohen’s kappa test. Kaplan–Meier curve analysis of prognosis was performed in patients with ICR and ECR and differences between curves were compared by log-rank test. The differences in vascular heterogeneity parameters between ICR and ECR were assessed using Student’s *t*-test. Bonferroni’s correction (*n* = 4) was applied for multiple comparisons of vascular heterogeneity parameters at four vascular habitats, and the adjusted significance level was *α* = 0.0125 (0.05/4 = 0.0125). Area under the receiver operating characteristic (ROC) curve (AUC) was used to analyze the diagnostic performance of vascular heterogeneity parameters in discriminating ICR and ECR. The optimal cut-off value of the parameters was derived using the Youden index. Variables with *p*-value < 0.05 from univariable logistic regression analysis were entered into the multivariable logistic regression analysis to identify the predictors of spatial recurrence pattern, to check the multicollinearity of the predictors, and to construct a nomogram model. A nomogram performance was evaluated using calibration curve and ROC. Internal validation of the nomogram model was performed using the bootstrap technique with 1000 repetitions to assess the predictive accuracy.

## Results

### Patient characteristics

**Fig. 2:**
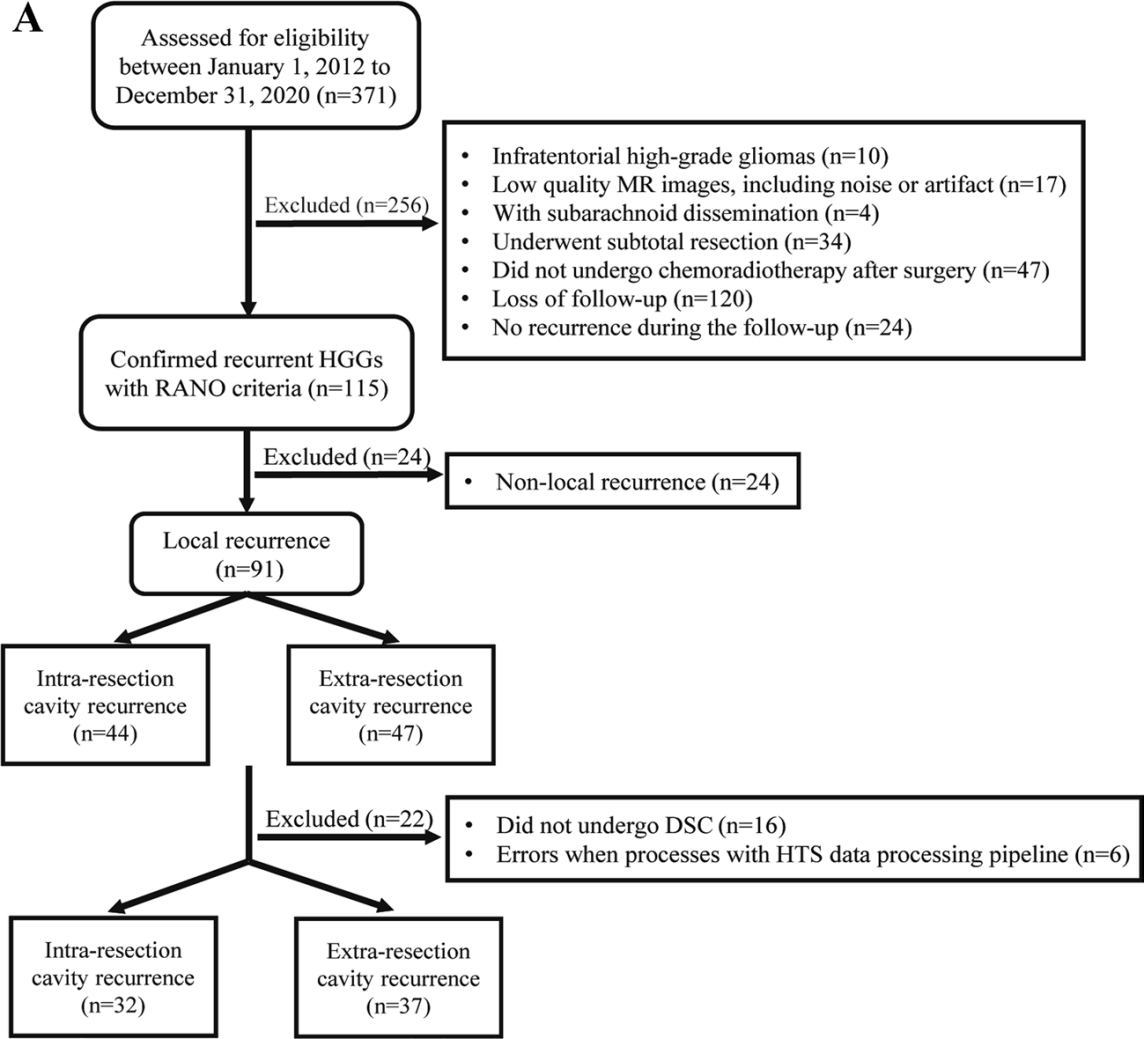

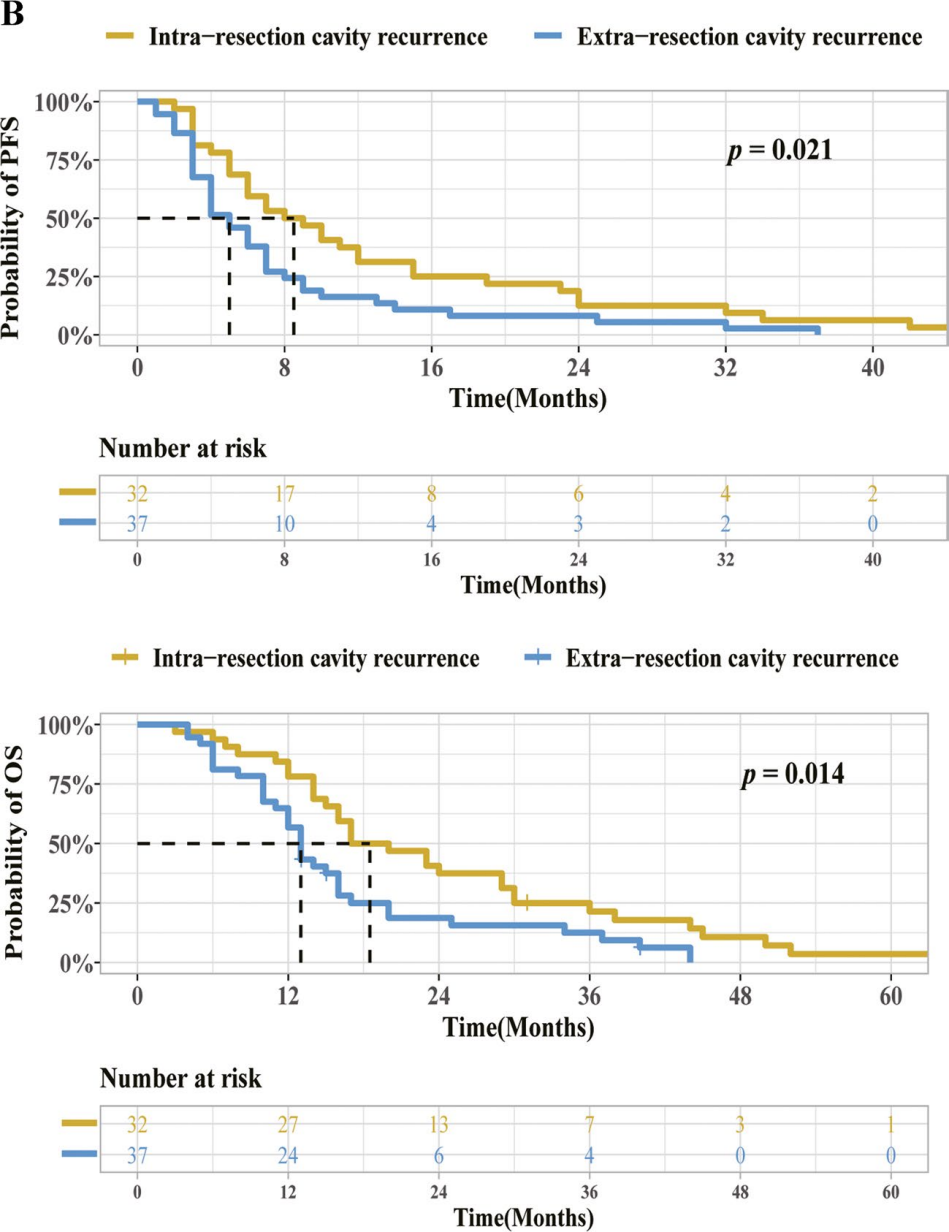
**A** The flow diagram of patient selection process. **B** Kaplan–Meier plots for PFS (top panel) and OS (bottom panel) comparing intra-resection cavity recurrence vs extra-resection cavity recurrence. Intra-resection cavity recurrence was associated with longer PFS (*p* = 0.021) and OS (*p* = 0.014). HGGs high-grade gliomas, RANO Response Assessment in Neuro-Oncology, DSC dynamic susceptibility contrast, HST hemodynamic tissue signature, PFS progression-free survival, OS overall survival

We identified 371 patients with HGGs who met the inclusion criteria. According to the exclusion criteria, 69 local recurrence patients (mean age, 50 ± 15 years, 35 female) were eventually included. The patient screening process is shown in Fig. 2A. The clinical and pathological characteristics and radiological features of the 69 patients with ICR (*n* = 32) and ECR (*n* = 37) are summarized in Table 1. The inter-rater reliability (Supplementary Table 1) between the neurosurgeon and neuroradiologist revealed near-perfect agreement for radiological features.

**Table 1:** Patients’ clinical and pathological characteristics and radiological features

	Intra-resection cavity recurrence (*n* = 32)	Extra-resection cavity recurrence (*n* = 37)	*p*-value
Sex: female/male	17/15	18/19	0.711 ^*a*^
Age (year) ^*^	48 ± 15	52 ± 14	0.168 ^*b*^
Pre-surgery KPS score ^*^	70 ± 14	70 ± 16	0.941 ^*b*^
WHO grade			
Grade III	16 (50.0)	7 (18.9)	0.006 ^*a*^
Grade IV	16 (50.0)	30 (81.1)	
IDH mutation status			
Mutant	3 (9.4)	2 (5.4)	0.667^*c*^
Wild-type	20 (62.5)	27 (73.0)	
Undetermined	9 (28.1)	8 (21.6)	
Tumor location			
Frontal	9 (28.1)	19 (51.4)	0.142 ^*c*^
Parietal	4 (12.5)	5 (13.5)	
Temporal	10 (31.3)	8 (21.6)	
Occipital	0 (0)	1 (2.7)	
Other	9 (28.1)	4 (10.8)	
SVZ involvement			
Yes	15 (46.9)	20 (54.1)	0.552 ^*a*^
No	17 (53.1)	15 (46.9)	
Cortex infiltrated			
Yes	18 (56.2)	23 (62.2)	0.618 ^*a*^
No	14 (43.8)	14 (37.8)	
Ventricular entry			
Yes	6 (18.7)	16 (43.2)	0.029 ^*a*^
No	26 (81.3)	21 (56.8)	

Data are numbers of patients and data in parentheses are percentages. ^*^Data are means ± SD. ^*a*^Pearson chi squared test, ^*b*^Student’s *t*-test, ^*c*^Fisher’s exact test. *KPS* Karnofsky Performance Scale, *IDH* isocitrate dehydrogenase, *SVZ* subventricular zone

### The PFS and OS are associated with spatial recurrence pattern

Kaplan–Meier survival analysis of 69 local recurrence patients indicated that the median PFS was 6 months, and the median OS was 16 months. There were significant differences in prognosis between the two spatial recurrence patterns (Fig. 2B). The median PFS was 8 months for ICR and 5 months for ECR. PFS was longer in ICR compared to that in ECR (log-rank test *p* = 0.021). The median OS was 17 months for ICR and 13 months for ECR. OS for ICR were longer than those for ECR (log-rank test *p* = 0.014).

### Differences in vascular heterogeneity parameters among two spatial recurrence patterns

**Fig. 3:**
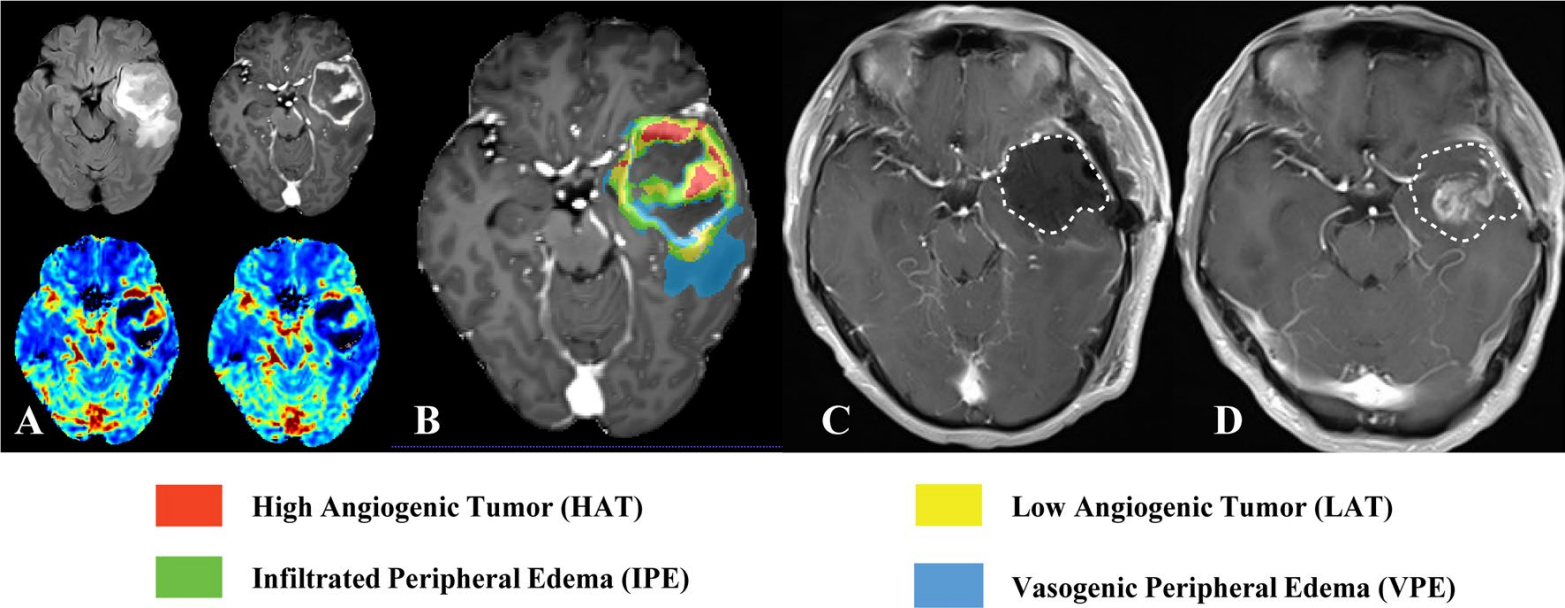
MRI scans in a 55-year-old man with glioblastoma, isocitrate dehydrogenase (IDH) wild-type, WHO grade IV, and tumor recurrence in the intra-resection cavity. Axial (**A**) fluid attenuation inversion recovery (FLAIR) image, contrast-enhanced T1-weighted image, cerebral blood volume (CBV) map, and cerebral blood flow (CBF) map are shown in small images (left to right, top to bottom). **B** Vascular habitat maps. The different colors indicate subregions of segmentation. **C** Twenty-four to 72 h postoperatively MRI. Contrast-enhanced T1-weighted image displayed gross total resection of the tumor and without enhancement of the surgical residual cavity (white dashed line). **D** Follow-up MRI. Contrast-enhanced T1-weighted image showed the tumor recurrence in the intra-resection cavity after 3 months

**Fig. 4:**
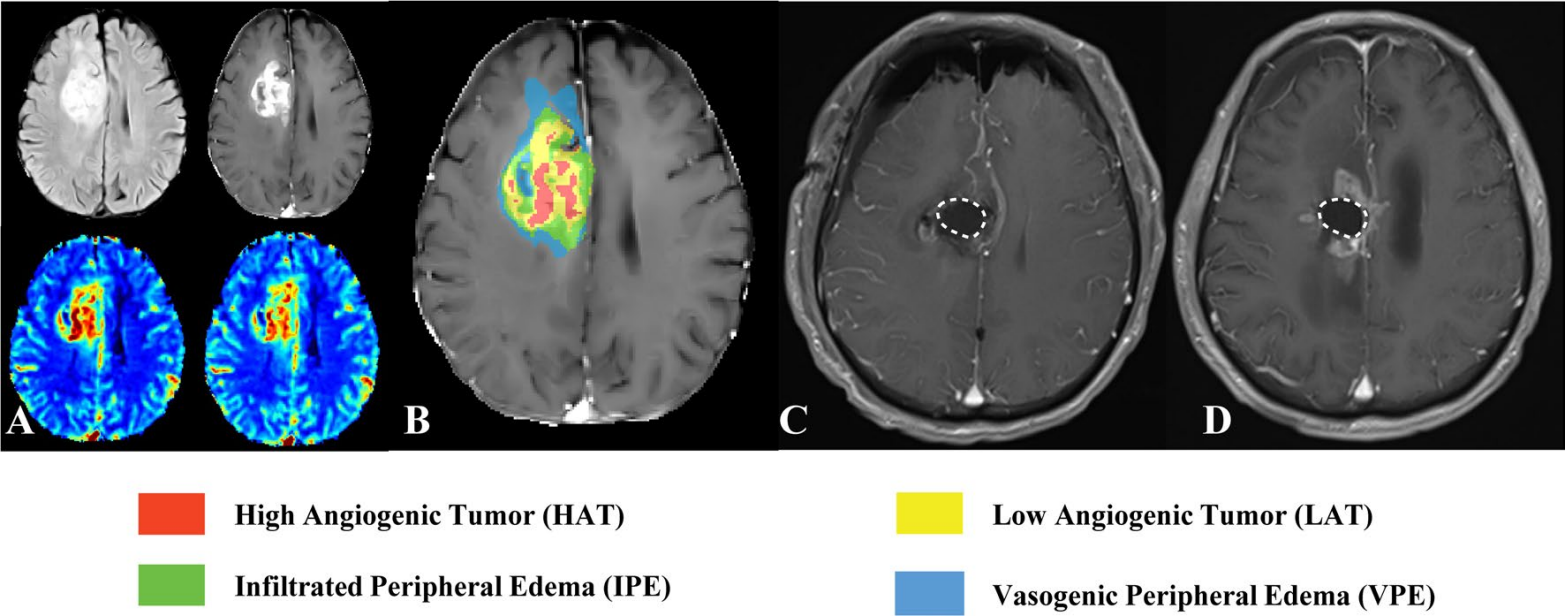
MRI scans in a 70-year-old man with glioblastoma, isocitrate dehydrogenase (IDH) wild-type, WHO grade IV, and tumor recurrence in the extra-resection cavity. Axial (**A**) fluid attenuation inversion recovery (FLAIR) image, contrast-enhanced T1-weighted image, cerebral blood volume (CBV) map, and cerebral blood flow (CBF) map are shown in small images (left to right, top to bottom). **B** Vascular habitat maps. The different colors indicate subregions of segmentation. **C** Twenty-four to 72 h postoperatively MRI. Contrast-enhanced T1-weighted image displayed gross total resection of the tumor and without enhancement of the surgical residual cavity (white dashed line). **D** Follow-up MRI. The tumor recurrence in the extra-resection cavity after 3 months

The four vascular habitats, HAT, LAT, IPE, and VPE, were segmented and labeled with different colors. Figures 3 and 4 show the vascular habitat maps, postoperative MRI, and follow-up MRI of two patients with ICR and ECR, respectively**.**

**Fig. 5:**
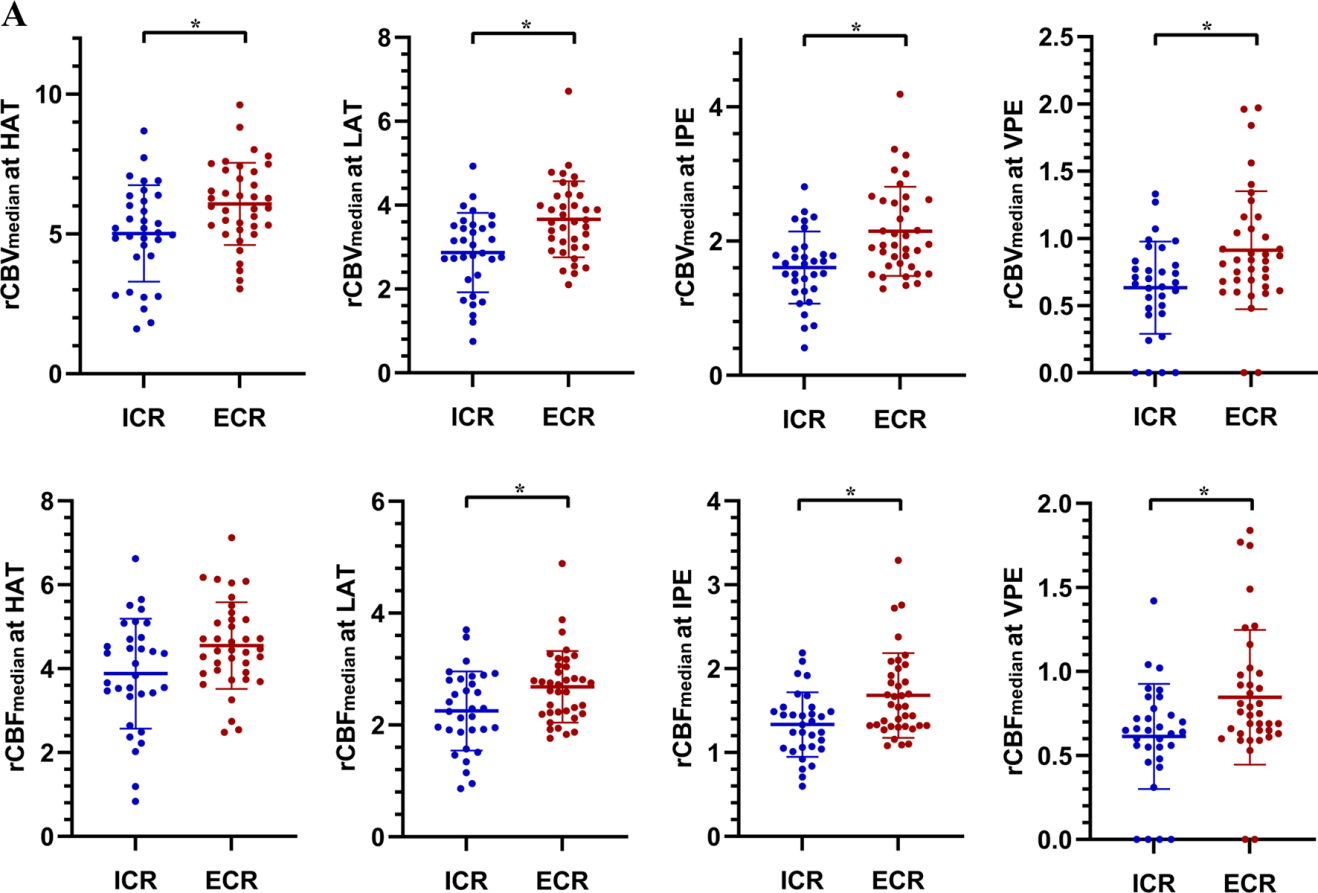

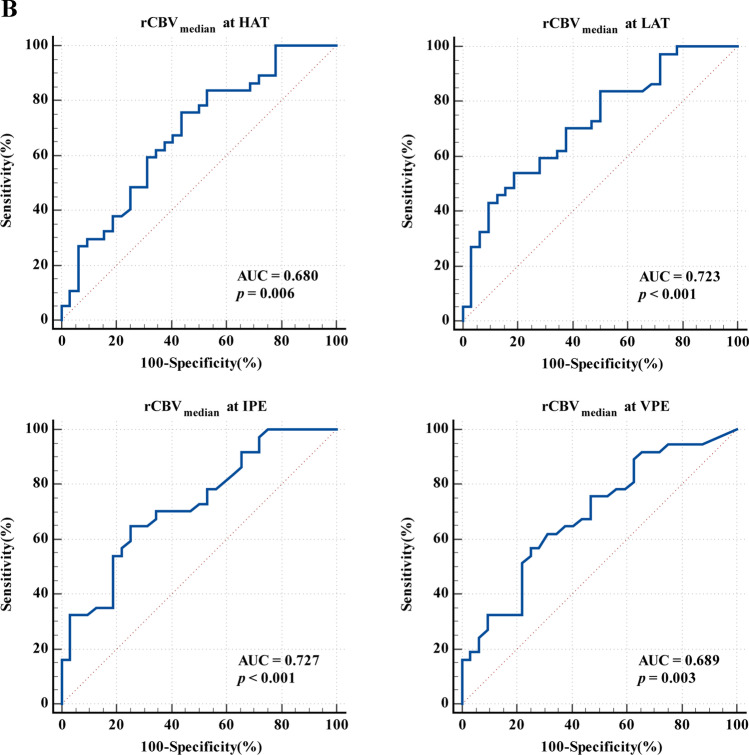
**A** Scatter plots of the rCBV_median_ at HAT, rCBV_median_ at LAT, rCBV_median_ at IPE, rCBV_median_ at VPE, rCBF_median_ at HAT, rCBF_median_ at LAT, rCBF_median_ at IPE, and rCBF_median_ at VPE (left to right, top to bottom) between intra- and extra-resection cavity recurrence. **B** Receiver operating characteristic (ROC) curves for differentiating intra- and extra-resection cavity recurrence. The area under the receiver operating characteristic curves (AUC) of the rCBV_median_ at HAT, rCBV_median_ at LAT, rCBV_median_ at IPE, and rCBV_median_ at VPE were 0.680 (95%CI: 0.557, 0.788, *p* = 0.006), 0.723 (95%CI: 0.603, 0.824, *p* < 0.001), 0.727 (95%CI: 0.607, 0.828, *p* < 0.001), and 0.689 (95%CI: 0.566, 0.793, *p* = 0.003), respectively. *Adjusted significance level *α* = 0.05/4 = 0.0125. rCBV_median_ the median relative cerebral blood volume, rCBF_median_ the median relative cerebral blood flow, HAT high angiogenic tumor, LAT low angiogenic tumor, IPE infiltrated peripheral edema, VPE vasogenic peripheral edema, ICR intra-resection cavity recurrence, ECR extra-resection cavity recurrence

The rCBV_median_ and rCBF_median_ for each vascular habitat were compared across the two spatial patterns (Fig. 5A). Notably, the rCBV_median_ values in HAT, LAT, IPE, and VPE of the ECR were 6.077 ± 1.467, 3.661 ± 0.907, 2.146 ± 0.665, and 0.912 ± 0.439, respectively. The rCBV_median_ values in HAT, LAT, IPE, and VPE of the ICR were 5.019 ± 1.724, 2.868 ± 0.946, 1.608 ± 0.537, and 0.633 ± 0.344, respectively. Student’s *t*-test indicated that the rCBV_median_ of HAT (*p* = 0.008), LAT (*p* = 0.001), IPE (*p* < 0.001), and VPE (*p* = 0.005) were higher in the ECR than in the ICR. The rCBF_median_ values in HAT, LAT, IPE, and VPE of the ECR were 4.550 ± 1.035, 2.683 ± 0.642, 1.681 ± 0.505, and 0.846 ± 0.401, respectively. The rCBF_median_ values in HAT, LAT, IPE, and VPE of the ICR were 3.881 ± 1.310, 2.250 ± 0.709, 1.334 ± 0.385, and 0.613 ± 0.313, respectively. Student’s *t*-test indicated that the rCBF_median_ of LAT (*p* = 0.010), IPE (*p* = 0.002), and VPE (*p* = 0.010) was higher in the ECR than in the ICR. There was no evidence of a difference in the rCBF_median_ of HAT between the ICR and ECR (*p* = 0.021). The rCBV_mean_, rCBF_mean_, rCBV_max_, and rCBF_max_ for each vascular habitat were compared across the two spatial patterns (Supplementary Table 2).

### Diagnostic performance of vascular heterogeneity parameters in evaluating spatial recurrence pattern

The diagnostic performance of the rCBV_median_ at each vascular habitat is shown in Fig. 5B and Supplementary Table 3. The AUC of rCBV_median_ at HAT to discriminate ICR from ECR was 0.680 (95%CI: 0.557, 0.788) and the optimal cut-off value was 5.2 (sensitivity: 75.68%, specificity: 56.25%). For the rCBV_median_ at LAT, the AUC was 0.723 (95%CI: 0.603, 0.824) and the optimal cut-off value was 3.53 (sensitivity: 54.05%, specificity: 81.25%). For the rCBV_median_ at IPE, the AUC was 0.727 (95%CI: 0.607, 0.828) and the optimal cut-off value was 1.8 (sensitivity: 64.86%, specificity: 75.00%). For the rCBV_median_ at VPE, the AUC was 0.689 (95%CI: 0.566, 0.795) and the optimal cut-off value was 0.8 (sensitivity: 56.76%, specificity: 75.00%). The diagnostic performance of the rCBV_median_ in each vascular habitat was good (*p* < 0.05). Additionally, the diagnostic performance of the rCBF_median_ at each vascular habitat is shown in Supplementary Fig. 1.

### Risk factor for spatial recurrence pattern

Three prediction models were constructed, model 1: median rCBV and rCBF (Supplementary Table 4), model 2: max rCBV and rCBF (Supplementary Table 5), and model 3: mean rCBV and rCBF (Supplementary Table 6). ROC curve analysis and bootstrapping verification results showed that model 1 had the optimal performance, and it was selected as the final model (Supplementary Table 7).

**Fig. 6:**
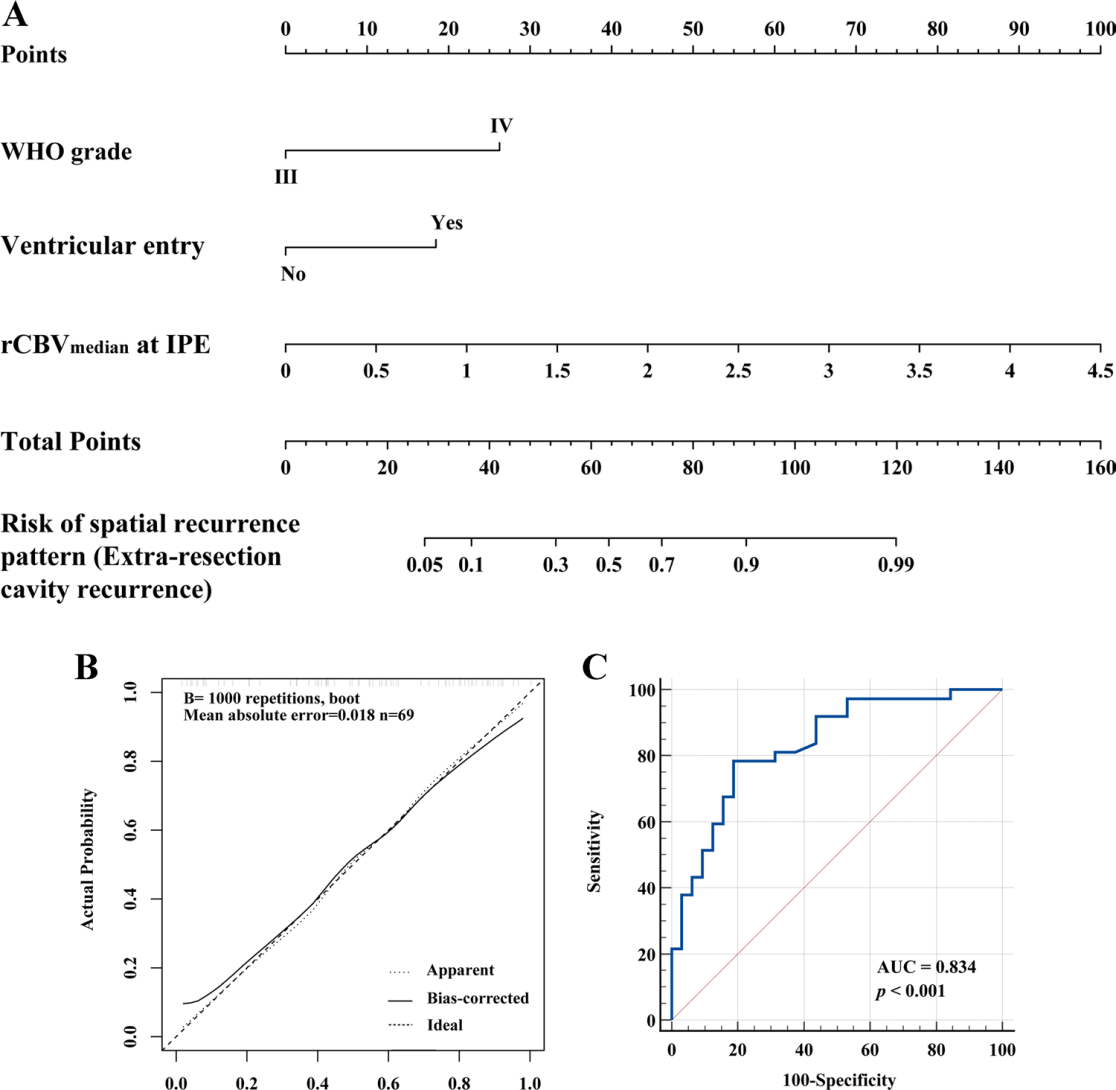
**A** A nomogram predicting the risk of extra-resection cavity recurrence. The value of WHO grade, ventricular entry, and rCBV_median_ at IPE was scored on the point scale axis. The probability of extra-resection cavity recurrence could be estimated by adding each score to calculate a total score, which is projected onto a total point scale. **B** The calibration curves for the nomogram model. The *x*-axis represents the nomogram-predicted probability and the *y*-axis represents the actual probability of extra-resection cavity recurrence. The diagonal dashed line represents a perfect prediction by an ideal model. The dotted line represents the entire cohort (*n* = 69), and the solid line is bias-corrected by bootstrapping (*B* = 1000). The closer the solid line is to the diagonal dashed line, the better prediction. **C** Receiver operating characteristic curves for discriminating intra- and extra-resection cavity recurrence. The area under the receiver operating characteristic curves (AUC) of the nomogram model was 0.834 (95%CI: 0.726, 0.913)

In the multivariable logistic regression analysis, WHO grade (OR: 8.486, 95%CI: 2.000, 36.008, *p* = 0.004), ventricular entry (OR: 4.492, 95%CI: 1.135, 17.770, *p* = 0.032), and rCBV_median_ at IPE (OR: 6.112, 95%CI: 1.793, 20.834, *p* = 0.004) were confirmed as risk factors for ECR. The multicollinearity test showed the variance inflation factors of WHO grade, ventricular entry, and rCBV_median_ at IPE were 1.018, 1.051, and 1.042, respectively, indicating that there was no multicollinearity among the independent factors. These independent factors were used to construct a nomogram model (Fig. 6A). Calibration curve and the nonsignificant Hosmer–Lemeshow test (*p* = 0.461) demonstrated the nomogram model had good calibration (Fig. 6B). The AUC of the nomogram to predict spatial recurrence pattern was 0.834 (95%CI: 0.726, 0.913; sensitivity: 78.38%; specificity: 81.25%) (Fig. 6C) and was confirmed as 0.833 (95%CI: 0.830, 0.836) after internal validation.

## Discussion

Although there are different spatial patterns in local recurrence, the prognosis between the different spatial recurrence patterns is unclear, and there is a lack of studies on whether imaging markers can predict recurrence pattern. Our study demonstrated that PFS and OS were longer in the ICR compared with those in the ECR. The rCBV_median_ at IPE could be used as an imaging marker to predict spatial pattern of local recurrence.

Local recurrence is the most common pattern in recurrent HGGs. In our study cohort, local recurrence accounted for 79.13% of recurrent HGGs under standard therapy. Local recurrence was divided into intra- and extra-resection cavity patterns, and patients with ICR had longer PFS and OS than those with ECR. MRI vascular habitat analysis demonstrated that preoperative vascular heterogeneity parameters of ECR were higher than those of ICR. Since all patients received standard therapy, the prognosis of ECR was shorter than that of ICR, indicating that high preoperative vascular heterogeneity was associated with poor prognosis. This result is consistent with previous literature [10, 11]. Alvarez-Torres et al found that rCBV reflected the degree of microvascular proliferation within the tumor [19]. Aggregation of tumor cells in areas with active microvascular proliferation results in a region that may exhibit higher heterogeneity. Glioma heterogeneity was associated with tumor recurrence [20, 21]. Our study showed the ECR exhibited higher heterogeneity compared with ICR, and that the vascular heterogeneity parameters had good diagnostic performance for distinguishing ICR and ECR.

In addition, tumor heterogeneity is not only limited to the tumor parenchyma but also involves the peritumoral area. Tumor cells have been considered to migrate from the core tumor region to the peritumoral brain region, and tumor recurrence has commonly been seen on imaging in the peritumoral edema area that is infiltrated by the tumor [22, 23]. Corresponding to our study, vascular habitat imaging of HGGs revealed that ECR had a high rCBV_median_ and rCBF_median_ at IPE. Despite the lack of pathological evidence, IPE indicated the area of tumor infiltration to some extent, and the residual tumor cells in this area after surgery might lead to ECR. Tumor infiltration was difficult to identify using conventional MRI, whereas vascular habitat imaging could initially segment the area of tumor infiltration.

In our study, we constructed a nomogram model based on WHO grade, ventricular entry, and rCBV_median_ at IPE. The AUC of the nomogram model was 0.834 and the fitting degree of the calibration curve was good. The rCBV_median_ at IPE can serve as an effective imaging marker to predict the spatial pattern in locally recurrent HGGs, providing a new diagnostic basis for patients with surgery and postoperative chemoradiotherapy. For patients with ECR, extended surgical resection might be considered, whereas additional doses of radiation therapy might be considered for those with ICR. The IPE may be a potential region of ECR in HGGs. It is worth exploring whether using IPE as the surgical resection range can improve the prognosis of patients in the future. Vascular heterogeneity parameters generated by vascular habitats not only contained prognosis information for patients [10, 11], but also predicted spatial recurrence patterns. In our study, vascular habitats were based on DSC to reflect vascular heterogeneity. However, multiparametric MRI can reflect the physiological information of tumors, including cellularity, vascularity, and tumor metabolism. The habitats constructed based on multimodal imaging can provide more information about intratumoral heterogeneity [24–26]. Previously, the habitat constructed by DSC and DWI quantitative parameters had a sensitivity of 77.1% and specificity of 89.4% in distinguishing tumor progression from treatment-related change, and predicted the progression site of post-treatment glioblastoma [27]. In addition, the habitat obtained by electrical properties tomography and physiological MRI could provide more comprehensive tissue-level information [28]. Therefore, the habitat derived from multiparametric physiological MRI may help to improve the effectiveness of predicting spatial recurrence patterns in locally recurrent HGGs, and further research on multiparametric habitat analysis is needed.

Our study had some limitations. Firstly, this was a single-center retrospective study. So, it is necessary to conduct a multicenter validation study and expand the cohort size in future studies. Secondly, the pathological molecular statues of some cases in the study cohort were incomplete. Previous studies have reported that the spatial recurrence pattern of gliomas is correlated with pathological molecular markers [7, 29]. Therefore, it is meaningful to explore the combination of vascular heterogeneity parameters and pathological molecular markers to improve the predictive performance of spatial recurrence pattern. In addition, although the nomogram model did not complete external validation, internal validation has confirmed the model has good performance (AUC = 0.833, 95%CI: 0.830, 0.836), laying a foundation for verification in external databases and clinical practice.

In conclusion, the spatial pattern of locally recurrent HGGs is associated with prognosis. The rCBV_median_ at IPE could be used as a non-invasive imaging marker to predict spatial recurrence pattern, providing a new diagnostic basis for clinicians to develop the extent of surgical resection and postoperative radiotherapy dose planning.

### Supplementary information

Below is the link to the electronic supplementary material.

Supplementary file1 (PDF 200 KB)

## Abbreviations

CE: Contrast-enhanced
DSC: Dynamic susceptibility contrast
ECR: Extra-resection cavity recurrence
FLAIR: Fluid-attenuated inversion recovery
FOV: Field of view
Gd-DTPA: Gadopentetate glucosamine
HAT: High angiogenic tumor
HGGs: High-grade gliomas
HTS: Hemodynamic tissue signature
ICR: Intra-resection cavity recurrence
IPE: Infiltrated peripheral edema
LAT: Low angiogenic tumor
OS: Overall survival
PFS: Progression-free survival
RANO: Response Assessment in Neuro-Oncology
rCBF: Relative cerebral blood flow
rCBV: Relative cerebral blood volume
SVZ: Subventricular zone
VPE: Vasogenic peripheral edema

## Declarations

### Guarantor

The scientific guarantor of this publication is Shunan Wang, M.D., Ph.D.

### Statistics and biometry

One of the authors (H.W.) has significant statistical expertise.

### Methodology

• Retrospective

• Experimental

• Performed at one institution

## References

[1] Ostrom QT, Cioffi G, Waite K, Kruchko C, Barnholtz-Sloan JS (2021). CBTRUS Statistical Report: primary brain and other central nervous system tumors diagnosed in the United States in 2014–2018. Neuro Oncol.

[2] Stupp R, Mason WP, van den Bent MJ (2005). Radiotherapy plus concomitant and adjuvant temozolomide for glioblastoma. N Engl J Med.

[3] Tan AC, Ashley DM, López GY, Malinzak M, Friedman HS, Khasraw M (2020). Management of glioblastoma: state of the art and future directions. CA Cancer J Clin.

[4] Chamberlain MC (2011). Radiographic patterns of relapse in glioblastoma. J Neurooncol.

[5] Rapp M, Baernreuther J, Turowski B, Steiger HJ, Sabel M, Kamp MA (2017). Recurrence pattern analysis of primary glioblastoma. World Neurosurg.

[6] Faustino AC, Viani GA, Hamamura AC (2020). Patterns of recurrence and outcomes of glioblastoma multiforme treated with chemoradiation and adjuvant temozolomide. Clinics (Sao Paulo).

[7] Jiang H, Yu K, Li M (2020). Classification of progression patterns in glioblastoma: analysis of predictive factors and clinical implications. Front Oncol.

[8] Pasqualetti F, Montemurro N, Desideri I (2022). Impact of recurrence pattern in patients undergoing a second surgery for recurrent glioblastoma. Acta Neurol Belg.

[9] Konishi Y, Muragaki Y, Iseki H, Mitsuhashi N, Okada Y (2012). Patterns of intracranial glioblastoma recurrence after aggressive surgical resection and adjuvant management: retrospective analysis of 43 cases. Neurol Med Chir (Tokyo).

[10] Juan-Albarracín J, Fuster-Garcia E, Pérez-Girbés A (2018). Glioblastoma: vascular habitats detected at preoperative dynamic susceptibility-weighted contrast-enhanced perfusion MR imaging predict survival. Radiology.

[11] Del Mar Á-T, Juan-Albarracín J, Fuster-Garcia E (2020). Robust association between vascular habitats and patient prognosis in glioblastoma: an international multicenter study. J Magn Reson Imaging.

[12] Fuster-Garcia E, Lorente Estellés D, Álvarez-Torres MDM (2021). MGMT methylation may benefit overall survival in patients with moderately vascularized glioblastomas. Eur Radiol.

[13] Álvarez-Torres MDM, Fuster-García E, Balaña C, Puig J, García-Gómez JM (2021). Lack of benefit of extending temozolomide treatment in patients with high vascular glioblastoma with methylated MGMT. Cancers (Basel).

[14] Álvarez-Torres MDM, Fuster-García E, Reynés G (2021). Differential effect of vascularity between long- and short-term survivors with IDH1/2 wild-type glioblastoma. NMR Biomed.

[15] Wu H, Tong H, Du X (2020). Vascular habitat analysis based on dynamic susceptibility contrast perfusion MRI predicts IDH mutation status and prognosis in high-grade gliomas. Eur Radiol.

[16] Chelebian E, Fuster-Garcia E, Álvarez-Torres MDM, Juan-Albarracín J, García-Gómez JM (2020). Higher vascularity at infiltrated peripheral edema differentiates proneural glioblastoma subtype. PLoS One.

[17] Juan-Albarracín J, Fuster-Garcia E, García-Ferrando GA, García-Gómez JM (2019). ONCOhabitats: a system for glioblastoma heterogeneity assessment through MRI. Int J Med Inform.

[18] Petrecca K, Guiot MC, Panet-Raymond V, Souhami L (2013). Failure pattern following complete resection plus radiotherapy and temozolomide is at the resection margin in patients with glioblastoma. J Neurooncol.

[19] Álvarez-Torres MDM, Fuster-García E, Juan-Albarracín J (2022). Local detection of microvessels in IDH-wildtype glioblastoma using relative cerebral blood volume: an imaging marker useful for astrocytoma grade 4 classification. BMC Cancer.

[20] Patel AP, Tirosh I, Trombetta JJ (2014). Single-cell RNA-Seq highlights intratumoral heterogeneity in primary glioblastoma. Science.

[21] Eder K, Kalman B (2014). Molecular heterogeneity of glioblastoma and its clinical relevance. Pathol Oncol Res.

[22] Lemée JM, Clavreul A, Menei P (2015). Intratumoral heterogeneity in glioblastoma: don’t forget the peritumoral brain zone. Neuro Oncol.

[23] Bastola S, Pavlyukov MS, Yamashita D (2020). Glioma-initiating cells at tumor edge gain signals from tumor core cells to promote their malignancy. Nat Commun.

[24] Lee J, Narang S, Martinez J, Rao G, Rao A (2015). Spatial habitat features derived from multiparametric magnetic resonance imaging data are associated with molecular subtype and 12-month survival status in glioblastoma multiforme. PLoS One.

[25] John F, Bosnyák E, Robinette NL (2019). Multimodal imaging-defined subregions in newly diagnosed glioblastoma: impact on overall survival. Neuro Oncol.

[26] Park JE, Kim HS, Kim N, Park SY, Kim YH, Kim JH (2021). Spatiotemporal heterogeneity in multiparametric physiologic MRI is associated with patient outcomes in IDH-wildtype glioblastoma. Clin Cancer Res.

[27] Kim M, Park JE, Kim HS (2021). Spatiotemporal habitats from multiparametric physiologic MRI distinguish tumor progression from treatment-related change in post-treatment glioblastoma. Eur Radiol.

[28] Park JE, Kim HS, Kim N (2021). Low conductivity on electrical properties tomography demonstrates unique tumor habitats indicating progression in glioblastoma. Eur Radiol.

[29] Ge S, Shi Y, Zhu G (2020). Molecular pathological markers correlated with the recurrence patterns of glioma. Front Oncol.

