# Supplementary material for: Preoperative vascular heterogeneity based on dynamic susceptibility contrast MRI in predicting spatial pattern of locally recurrent high-grade gliomas
Source: Eur Radiol. 2023 Sep 2;34(3):1982–93. doi: 10.1007/s00330-023-10149-6 (PMC10873240; doi:10.1007/s00330-023-10149-6)
Supplement: Supplementary file 1 — Supplementary file1 (PDF 200 KB) [file 330_2023_10149_MOESM1_ESM.pdf]

Preoperative vascular heterogeneity based on dynamic susceptibility contrast MRI in predicting spatial pattern of locally recurrent high-grade gliomas

Electronic Supplementary Material

Eur Radiol (2023) Wang H, Zeng L, Wu H et al.

Figures

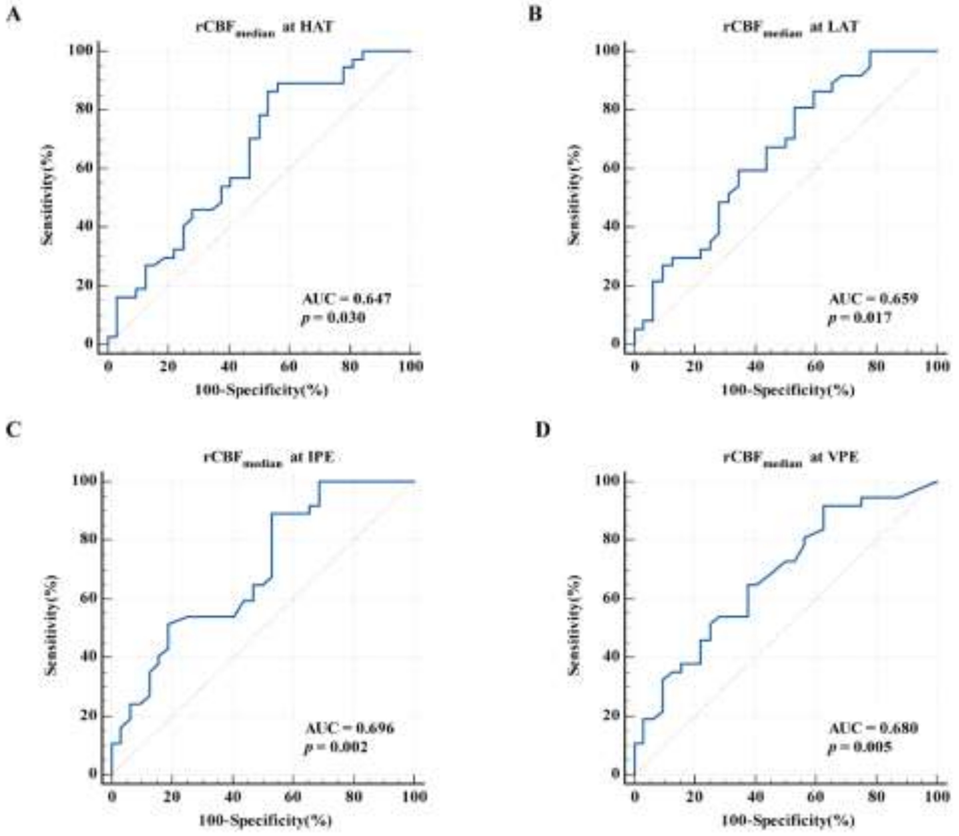

Supplementary Figure 1. Receiver operating characteristic (ROC) curves for differentiating intra- and extra-resection cavity recurrence. The area under the receiver operating characteristic curves (AUC) of the $rCBF_{median}$ at HAT, $rCBF_{median}$ at LAT, $rCBF_{median}$ at IPE, and $rCBF_{median}$ at VPE were 0.647 (95%CI: 0.523, 0.759, p=0.030), 0.659 (95%CI: 0.535, 0.769, p=0.017), 0.696 (95%CI: 0.574, 0.801, p=0.002), 0.680 (95%CI: 0.557, 0.787, p=0.005), respectively. $rCBF_{median}$ = the median relative cerebral blood flow, HAT = high angiogenic tumor, LAT = low angiogenic tumor, IPE = infiltrated peripheral edema, VPE = vasogenic peripheral edema.

Tables

Supplementary Table 1. Interrater reliability analysis (Cohen's kappa coefficient)

	A neurosurgeon and neuroradiologist	95%CI
Spatial recurrence pattern	0.882	0.770, 0.994
SVZ involvement	0.855	0.733, 0.977
Cortex infiltrated	0.852	0.729, 0.975
Ventricular entry	0.899	0.787, 1.011

Cohen's kappa coefficient: 1 = perfect agreement, 0.81–0.99 = near perfect agreement, 0.61–0.80 = substantial agreement, 0.41–0.60 = moderate agreement, 0.21–0.40 = fair agreement, 0.1–0.20 = slight agreement, 0 = agreement equivalent to chance. SVZ = subventricular zone.

Supplementary Table 2. Differences in vascular heterogeneity parameters among two spatial recurrence patterns

	intra-resection cavity recurrence	extra-resection cavity recurrence	p
$rCBV_{median}$ at HAT	5.019 ± 1.724	6.077 ± 1.467	0.008*
$rCBF_{median}$ at HAT	3.881 ± 1.310	4.550 ± 1.035	0.021
$rCBV_{median}$ at LAT	2.868 ± 0.946	3.661 ± 0.907	0.001*
$rCBF_{median}$ at LAT	2.250 ± 0.709	2.683 ± 0.642	0.010*
$rCBV_{median}$ at IPE	1.608 ± 0.537	2.146 ± 0.665	< 0.001*
$rCBF_{median}$ at IPE	1.334 ± 0.385	1.681 ± 0.505	0.002*
$rCBV_{median}$ at VPE	0.633 ± 0.344	0.912 ± 0.439	0.005*
$rCBF_{median}$ at VPE	0.613 ± 0.313	0.846 ± 0.401	0.010*
$rCBV_{max}$ at HAT	7.436 ± 1.886	7.642 ± 1.137	0.579
$rCBF_{max}$ at HAT	6.675 ± 2.226	6.948 ± 1.094	0.511
$rCBV_{max}$ at LAT	4.964 ± 1.671	6.262 ± 1.425	0.001*
$rCBF_{max}$ at LAT	4.112 ± 1.327	4.870 ± 1.181	0.014
$rCBV_{max}$ at IPE	2.924 ± 0.989	3.738 ± 1.065	0.002*
$rCBF_{max}$ at IPE	2.417 ± 0.683	2.849 ± 0.691	0.011*
$rCBV_{max}$ at VPE	1.393 ± 0.862	2.021 ± 1.116	0.012*
$rCBF_{max}$ at VPE	1.246 ± 0.689	1.658 ± 0.799	0.026
$rCBV_{mean}$ at HAT	5.089 ± 1.716	5.896 ± 1.442	0.037
$rCBF_{mean}$ at HAT	4.209 ± 1.475	4.580 ± 1.062	0.231

$rCBV_{mean}$ at LAT	2.695 ± 0.887	3.415 ± 0.923	0.002*
$rCBF_{mean}$ at LAT	2.223 ± 0.733	2.559 ± 0.671	0.055
$rCBV_{mean}$ at IPE	1.618 ± 0.538	2.120 ± 0.681	0.001*
$rCBF_{mean}$ at IPE	1.375 ± 0.431	1.699 ± 0.511	0.006*
$rCBV_{mean}$ at VPE	0.667 ± 0.328	0.972 ± 0.419	0.001*
$rCBF_{mean}$ at VPE	0.658 ± 0.332	0.908 ± 0.389	0.006*

* Adjusted significance level $\alpha = 0.0125$ ($0.05/4 = 0.0125$).

$rCBV_{median}$ = the median relative cerebral blood volume, $rCBF_{median}$ = the median relative cerebral blood flow, $rCBV_{max}$ = the max relative cerebral blood volume, $rCBF_{max}$ = the max relative cerebral blood flow, $rCBV_{mean}$ = the mean relative cerebral blood volume, $rCBF_{mean}$ = the mean relative cerebral blood flow

Supplementary Table 3. Diagnostic performance of the vascular heterogeneity parameters in differentiating intra- and extra-resection cavity recurrence

Parameter	Cut-off	Sensitivity	Specificity	AUC (95%CI)	*p* value
$rCBV_{median}$ at HAT	5.2	75.68	56.25	0.680 (0.557, 0.788)	0.006
$rCBV_{median}$ at LAT	3.53	54.05	81.25	0.723 (0.603, 0.824)	<0.001
$rCBV_{median}$ at IPE	1.8	64.86	75.00	0.727 (0.607, 0.828)	<0.001
$rCBV_{median}$ at VPE	0.8	56.76	75.00	0.689 (0.566, 0.795)	0.003
$rCBF_{median}$ at HAT	3.66	86.49	46.88	0.647 (0.523, 0.759)	0.030
$rCBF_{median}$ at LAT	2.15	81.08	46.88	0.659 (0.535, 0.769)	0.017
$rCBF_{median}$ at IPE	1.24	89.19	46.88	0.696 (0.574, 0.801)	0.002
$rCBF_{median}$ at VPE	0.56	91.89	37.50	0.680 (0.557, 0.787)	0.005

AUC = area under the receiver operating characteristic curve, $rCBV_{median}$ = the median relative cerebral blood volume, $rCBF_{median}$ = the median relative cerebral blood flow, HAT = high angiogenic tumor, LAT = low angiogenic tumor, IPE = infiltrated peripheral edema, VPE = vasogenic peripheral edema. The cut-off value was determined using Youden index.

Supplementary Table 4. Logistic regression analysis of spatial recurrence pattern (**Model 1: median rCBV and rCBF**)

Variable	Univariable		Multivariable	
	OR (95%CI)	*p* value	OR (95%CI)	*p* value
WHO grade	4.286 (1.462, 12.565)	**0.008**	**8.486 (2.000, 36. 008)**	**0.004**
IDH mutation status	0.916 (0.379, 2.211)	0.845		
SVZ involvement	1.333 (0.516, 3.443)	0.552		
Cortex infiltrated	1.278 (0.487, 3.350)	0.618		
Ventricular entry	3.302 (1.098, 9.923)	**0.033**	**4.492 (1.135, 17.770)**	**0.032**
$rCBV_{median}$ at HAT	1.536 (1.098, 2.147)	**0.012**		
$rCBV_{median}$ at LAT	2.764 (1.430, 5.342)	**0.002**		
$rCBV_{median}$ at IPE	5.218 (1.817, 14.988)	**0.002**	**6.112 (1.793, 20.834)**	**0.004**
$rCBV_{median}$ at VPE	6.953 (1.574, 30.721)	**0.011**		
$rCBF_{median}$ at HAT	1.659 (1.059, 2.600)	**0.027**		
$rCBF_{median}$ at LAT	2.779 (1.221, 6.326)	**0.015**		
$rCBF_{median}$ at IPE	7.109 (1.767, 28.600)	**0.006**		
$rCBF_{median}$ at VPE	7.161 (1.399, 36.662)	**0.018**		

OR = odd ratio, CI = confidence interval, SVZ = subventricular zone, $rCBV_{median}$ = the median relative cerebral blood volume, $rCBF_{median}$ = the median relative cerebral blood flow, HAT = high angiogenic tumor, LAT = low angiogenic tumor, IPE = infiltrated peripheral edema, VPE = vasogenic peripheral edema.

Supplementary Table 5. Logistic regression analysis of spatial recurrence pattern (**Model 2: max rCBV and rCBF**)

Variable	Univariable		Multivariable	
	OR (95%CI)	*p* value	OR (95%CI)	*p* value
WHO grade	4.286 (1.462, 12.565)	**0.008**	**5.158 (1.378, 19.309)**	**0.015**
IDH mutation status	0.916 (0.379, 2.211)	0.845		
SVZ involvement	1.333 (0.516, 3.443)	0.552		
Cortex infiltrated	1.278 (0.487, 3.350)	0.618		
Ventricular entry	3.302 (1.098, 9.923)	**0.033**	**3.930 (1.053, 14.659)**	**0.042**
$rCBV_{max}$ at HAT	1.095 (0.798, 1.504)	0.574		
$rCBV_{max}$ at LAT	1.752 (1.213, 2.532)	**0.003**	**1.564 (1.058, 2.311)**	**0.025**
$rCBV_{max}$ at IPE	2.336 (1.294, 4.218)	**0.005**		
$rCBV_{max}$ at VPE	2.046 (1.128, 3.710)	**0.018**		
$rCBF_{max}$ at HAT	1.101 (0.829, 1.461)	0.507		
$rCBF_{max}$ at LAT	1.676 (1.080, 2.601)	**0.021**		
$rCBF_{max}$ at IPE	2.763 (1.194, 6.398)	**0.018**		
$rCBF_{max}$ at VPE	2.208 (1.059, 4.606)	**0.035**		

OR = odd ratio, CI = confidence interval, SVZ = subventricular zone, $rCBV_{max}$ = the max relative cerebral blood volume, $rCBF_{max}$ = the max relative cerebral blood flow, HAT = high angiogenic tumor, LAT = low angiogenic tumor, IPE = infiltrated peripheral edema, VPE = vasogenic peripheral edema.

Supplementary Table 6. Logistic regression analysis of spatial recurrence pattern **(Model 3: mean rCBV and rCBF)**

Variable	Univariable		Multivariable	
	OR (95%CI)	*p* value	OR (95%CI)	*p* value
WHO grade	4.286 (1.462, 12.565)	**0.008**	**7.601 (1.904, 30.343)**	**0.004**
IDH mutation status	0.916 (0.379, 2.211)	0.845		
SVZ involvement	1.333 (0.516, 3.443)	0.552		
Cortex infiltrated	1.278 (0.487, 3.350)	0.618		
Ventricular entry	3.302 (1.098, 9.923)	**0.033**	**4.515 (1.163, 17.525)**	**0.029**
$rCBV_{mean}$ at HAT	1.399 (1.010, 1.939)	**0.044**		
$rCBV_{mean}$ at LAT	2.655 (1.368, 5.153)	**0.004**		
$rCBV_{mean}$ at IPE	4.306 (1.606, 11.548)	**0.004**	**4.678 (1.525, 14.351)**	**0.007**
$rCBV_{mean}$ at VPE	10.957 (2.062, 58.230)	**0.005**		
$rCBF_{mean}$ at HAT	1.267 (0.861, 1.864)	0.230		
$rCBF_{mean}$ at LAT	2.033 (0.966, 4.277)	0.062		
$rCBF_{mean}$ at IPE	5.000 (1.429, 17.489)	**0.012**		
$rCBF_{mean}$ at VPE	7.952 (1.581, 39.994)	**0.012**		

OR = odd ratio, CI = confidence interval, SVZ = subventricular zone, $rCBV_{mean}$ = the mean relative cerebral blood volume, $rCBF_{mean}$ = the mean relative cerebral blood flow, HAT = high angiogenic tumor, LAT = low angiogenic tumor, IPE = infiltrated peripheral edema, VPE = vasogenic peripheral edema.

Supplementary Table 7. The performance of 3 model in predicting the spatial recurrence pattern in locally recurrent HGGs.

Model	AUC (95%CI)	Sensitivity	Specificity	Bias-corrected AUC (95%CI)
Model 1(median)	**0.834 (0.726, 0.913)**	**78.38%**	**81.25%**	**0.833 (0.830, 0.836)**
Model 2(max)	0.797 (0.683, 0.885)	48.65%	96.87%	0.798 (0.794, 0.801)
Model 3(mean)	0.820 (0.709, 0.902)	72.97%	81.25%	0.821 (0.818, 0.824)

AUC = area under the receiver operating characteristic curve, CI = confidence interval.
